# CECS-CLIP: Fusing Domain Knowledge for Rare Wildlife Detection Model

**DOI:** 10.3390/ani14192909

**Published:** 2024-10-09

**Authors:** Feng Yang, Chunying Hu, Aokang Liang, Sheng Wang, Yun Su, Fu Xu

**Affiliations:** 1 School of Information Science and Technology, Beijing Forestry University, Beijing 100083, China; fengyang@bjfu.edu.cn (F.Y.); hhcyest123@bjfu.edu.cn (C.H.); bjfulak@bjfu.edu.cn (A.L.); ws13121192200@bjfu.edu.cn (S.W.); suyun0824@bjfu.edu.cn (Y.S.); 2 Engineering Research Center for Forestry-Oriented Intelligent Information Processing, National Forestry and Grassland Administration, Beijing 100083, China; 3 State Key Laboratory of Efficient Production of Forest Resources, Beijing 100083, China

**Keywords:** rare wildlife detection, multimodal learning, concept enhancement, feature scaling

## Abstract

**Simple Summary:**

Accurate detection of wildlife, particularly small and hidden animals, is crucial for conservation efforts. Traditional image-based methods often struggle in complex environments. This study introduces a novel approach that combines image and text data to improve detection accuracy. By incorporating textual information about animal characteristics and leveraging a Concept Enhancement Module (CEM), our model can better understand and locate animals, even in challenging conditions. Experimental results demonstrate a significant improvement in detection accuracy, achieving an average precision of 95.8% on a challenging wildlife dataset. Compared to existing multimodal target detection algorithms, this model achieved at least a 25% improvement in AP and excelled in detecting small targets of certain species, significantly surpassing existing multimodal target detection model benchmarks. This represents a substantial improvement compared to existing state-of-the-art methods. Our multimodal approach offers a promising solution for enhancing wildlife monitoring and conservation efforts.

**Abstract:**

Accurate and efficient wildlife monitoring is essential for conservation efforts. Traditional image-based methods often struggle to detect small, occluded, or camouflaged animals due to the challenges posed by complex natural environments. To overcome these limitations, an innovative multimodal target detection framework is proposed in this study, which integrates textual information from an animal knowledge base as supplementary features to enhance detection performance. First, a concept enhancement module was developed, employing a cross-attention mechanism to fuse features based on the correlation between textual and image features, thereby obtaining enhanced image features. Secondly, a feature normalization module was developed, amplifying cosine similarity and introducing learnable parameters to continuously weight and transform image features, further enhancing their expressive power in the feature space. Rigorous experimental validation on a specialized dataset provided by the research team at Northwest A&F University demonstrates that our multimodal model achieved a 0.3% improvement in precision over single-modal methods. Compared to existing multimodal target detection algorithms, this model achieved at least a 25% improvement in AP and excelled in detecting small targets of certain species, significantly surpassing existing multimodal target detection model benchmarks. This study offers a multimodal target detection model integrating textual and image information for the conservation of rare and endangered wildlife, providing strong evidence and new perspectives for research in this field.

## 1. Introduction

The precipitous decline in global biodiversity, primarily attributable to habitat loss, climate change, and anthropogenic pressures, necessitates urgent and evidence-based conservation strategies [1,2]. Wildlife monitoring emerges as a foundational component of these efforts, providing critical data on population dynamics, distribution, and behavior to inform targeted conservation interventions [3,4,5,6].

Traditional wildlife monitoring has been predominantly reliant on labor-intensive methods such as direct observation [7], transect surveys [8], and mark–recapture [9]. These approaches are often constrained by limitations in data accuracy and reliability due to observer bias and potential disturbance to wildlife behavior [10]. The integration of computer vision algorithms with unmanned aerial vehicle (UAV) and satellite imagery has ushered in a new paradigm for wildlife monitoring [11]. This technological advancement enables automated species detection, identification, and tracking across expansive geographic areas [12], providing unprecedented insights into population dynamics, behavior, and habitat use [13].

Conventional computer vision algorithms for wildlife monitoring primarily rely on single-image modalities, employing large-scale annotated datasets to train one-stage (YOLO, SSD) [14,15,16] or two-stage (Faster R-CNN) detectors [17,18]. Akito Takeki et al. [19] combined deep features from FCNs and DeepLab for semantic segmentation, employing a support vector machine to effectively detect objects of varying scales in large-scale imagery. Mirka et al. [20] leveraged thermal infrared (TIR) imagery captured by drones, incorporating contextual information such as thermal contrast and repeated site imaging (RSI) to accurately monitor arboreal monkey populations. Povlsen et al. [21] utilized drone-based thermal imaging video data, incorporating image enhancement techniques and negative samples to improve the detection of wildlife species such as hares and deer. Vega et al. [22] optimized the performance of ladybug target detection by experimenting with different distance metrics in the WHD loss function, combined with the U-Net network architecture and hyperparameter tuning.

While effective in controlled environments, these methods often struggle in real-world scenarios due to challenges [23] such as small object sizes, occlusions, and varying environmental conditions commonly encountered in remote sensing imagery [24,25]. The paucity of discriminative information within images of small, occluded, or camouflaged targets poses significant challenges for accurate wildlife monitoring; for instance, detecting small birds in dense foliage due to the lack of distinctive visual features. Moreover, environmental factors such as varying lighting conditions, atmospheric disturbances, and complex backgrounds can further exacerbate these challenges. Consequently, relying solely on visual data limits the effectiveness of traditional image-based detection methods, necessitating the exploration of complementary data sources to enhance detection accuracy and robustness [26].

Textual data, containing semantic information about animal attributes, behaviors, and habitats, can aid in target localization and identification [27]. This is especially beneficial in complex natural environments where wildlife exhibits high diversity and complexity, as textual data provide valuable prior knowledge that can guide the model towards the target [28,29]. The alignment of image and text modalities remains a challenging problem in multimodal learning due to the semantic gap between them [30]. Developing robust alignment mechanisms is essential for integrating information from different modalities.

To address the limitations of traditional image-based object detection methods in detecting small, occluded, or camouflaged wildlife, this study proposes a novel multimodal framework that leverages the complementary strengths of visual and textual information. By incorporating semantic information about animal attributes, behaviors, and habitats, the framework enhances the model’s semantic understanding of the visual scene. To effectively align visual and textual modalities, a Concept Enhancement Module (CEM) is integrated, employing cross-attention to correlate image and text features. Additionally, a feature normalization module is introduced to amplify subtle visual cues, enabling the model to better discriminate between target and background objects. This multimodal approach, coupled with the enhanced feature representation, offers a robust solution for wildlife conservation and surveillance in challenging scenarios.

Our framework incorporates several key innovations: (1) We propose a novel detection framework that significantly enhances the model’s understanding of complex visual scenes by integrating textual and visual information. Notably, our model excels at detecting objects that are small in size or partially occluded, a challenge that traditional methods struggle to address. (2) We introduce a continuous feature scaling method along with a learnable contrast parameter, applying continuous weighted transformations to image features to further strengthen their representational power in the feature space, enhancing the recognition accuracy of targets that are difficult to confirm solely through visual information due to their small size or partial occlusion. (3) We introduce an innovative Concept Enhancement Module (CEM) that significantly boosts the model’s semantic understanding capabilities. The CEM uses cross-attention to effectively align visual and textual features, enabling the model to capture more discriminative representations and improve object localization and recognition. (4) Compared to previous methods, we achieved a precision of 95.2% on the rare wildlife dataset, representing a 0.3% improvement over the original methods. Compared to existing multimodal object detection algorithms, this model achieved at least a 25% improvement in average precision.

## 2. Related Work

Deep learning is currently a hot topic. Zong et al. proposed an intelligent framework based on deep learning, which includes a Dynamic Attention Generative Adversarial Network (DATGAN) and a Parallel Spatial–Temporal Transformer (PSTTransformer) for missing data imputation and short-term prediction of traffic flow, reducing the mean square error by 5% in the data imputation task [31]. Liu et al. presented an automatic segmentation method combining a mask R-CNN and DBSCAN clustering algorithm for segmenting overlapped poplar seedling leaves under heavy metal stress [32]. Chen et al. introduced a multi-objective reinforcement learning approach named MORL-Trip for trip recommendation, aiming to provide personalized itinerary planning services for tourists in unfamiliar cities, constructing a series of ordered Points of Interest (POIs) to maximize travel experience with temporal and spatial constraints, as well as dynamic user preferences [33]. Nan et al. proposed a real-time lightweight object detection framework called HyperYOLO for processing multimodal remote sensing imagery, addressing the challenge of real-time processing of multimodal data on resource-constrained devices [34]. Yang et al. presented a fine-grained plant recognition network named PlantNet based on transfer learning and bilinear convolutional neural networks for high-throughput plant species identification in phenotyping analysis [35].

### 2.1. Single-Modal Object Detection

Single-modal object detection algorithms are primarily categorized into two-stage and one-stage detectors. Two-stage methods, such as R-CNNs and their variants, generate region proposals before classification and bounding box refinement [36]. While precise, these methods are computationally demanding [37,38,39]. One-stage detectors, exemplified by SSD and YOLO, directly predict object classes and locations in a single network pass, offering faster inference speeds at the potential cost of accuracy [40,41,42]. The latter’s speed is advantageous for applications like wildlife monitoring, where real-time detection is often crucial.

### 2.2. Multi-Modal Object Detection

Recent advancements in object detection have explored the potential of multimodal information [43]. Xu et al. introduced MQ-Det, a framework combining text and image modalities for enhanced object detection [44]. By leveraging the complementary strengths of both modalities, MQ-Det achieves improved detection accuracy for real-world targets. Zhong et al. proposed RegionCLIP, aligning region and text features through a pre-trained model, enabling precise object localization based on textual descriptions [45]. Mengjun et al. introduced ViSTA, a cross-modal retrieval algorithm that fuses image and text features at both local and global levels [46]. Finally, the GLIP model aligns regional and linguistic features through dot product operations, achieving effective object detection and phrase localization [47]. These studies collectively demonstrate the power of multimodal fusion for object detection, particularly in challenging scenarios such as wildlife monitoring.

### 2.3. Wildlife Monitoring

Recent advancements in object detection have yielded innovative approaches for wildlife monitoring. Roy et al. introduced WilDect-YOLO, a real-time model specifically designed for endangered species detection [48]. This work demonstrated the potential of deep learning for accurate wildlife identification in complex environments. Verma and Gupta focused on wildlife detection from camera trap image sequences, addressing challenges posed by dynamic backgrounds [49]. Eikelboom et al. applied deep learning to detect elephants, giraffes, and zebras in aerial imagery, showcasing the method’s applicability to large-scale monitoring [50]. These studies collectively highlight the growing importance of deep learning in addressing wildlife conservation challenges.

## 3. Materials and Methods

### 3.1. Dataset

**Figure 1:**
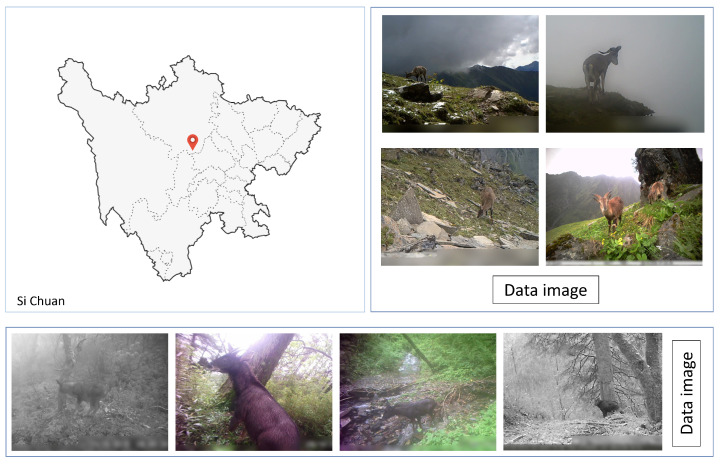
Spatial distribution of key protected areas within Sichuan Wolong National Nature Reserve.

In this study, we utilized a dataset focused on the detection of endangered wildlife to assess and experiment with the performance of our system. The dataset encompass the Wolong National Nature Reserve in the southwestern region of China’s Sichuan province, as well as the adjacent Mabian Dafengding Nature Reserve. It includes 28,000 images of 11 endangered wildlife species, such as the giant panda and the yellow-throated marten. These images capture a variety of scenes across different ecological seasons under fluctuating weather conditions and within diverse habitats. The dataset not only documents the appearance of the same species at various life stages but also the changes in imagery at the same location over different time points. The dataset was divided into 70% for the training set, 15% for the validation set, and 15% for the test set, ensuring sufficient data for model training and validity for evaluation. Additionally, a small dataset constructed from images collected from the internet was utilized to train and validate the target detection model for the same species to assess its performance in practical application scenarios. Figure 1 illustrates some data from the dataset.

### 3.2. CECS-CLIP Network

**Figure 2:**
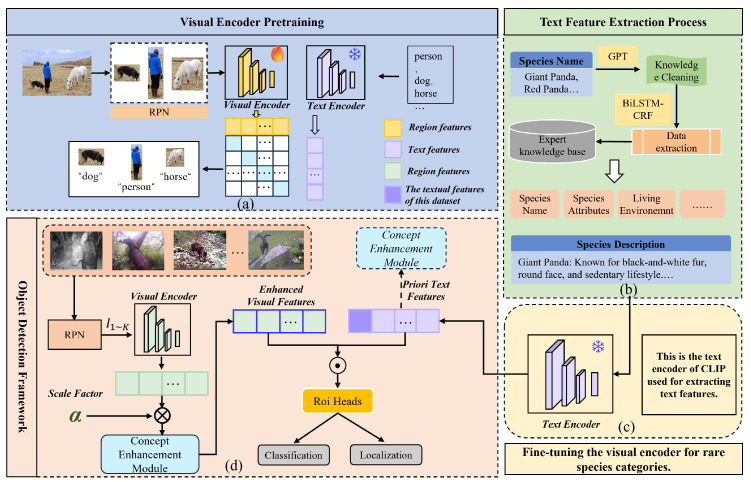
The overall framework of CECS-CLIP. (**a**) Pre-training of the visual encoder. CLIP is utilized to match images with descriptions, employing contrastive learning to extract visual region representations from generated image–text pairs. (**b**) Text feature extraction process. After generating textual information, data cleaning is performed, and textual knowledge is extracted through a BiLSTM-CRF model to construct an expert knowledge base. (**c**) Species description processing. Species descriptions are input into the text encoder to extract key textual features. (**d**) Joint application of visual and textual encoders. The well-trained visual encoder is integrated with the textual encoder for the object detection task.

This study introduces a novel multi-modal object detection framework, CECS-CLIP, tailored for the challenging task of rare wildlife monitoring. To address the limitations of traditional image-based methods in detecting small, occluded, or camouflaged animals, we integrate textual information to enrich the model’s understanding of the visual scene. Our framework leverages an advanced Vision-and-Language (ViL) model, incorporating a concept enhancement module to effectively fuse visual and textual features, as shown in Figure 2.

The proposed framework consists of two primary components: visual and textual representations. For textual representation, species names are extracted from the dataset and input into a pre-trained GPT model to generate detailed textual descriptions. These descriptions are then cleaned and transformed into a structured knowledge base using feature extraction techniques. The resulting knowledge base serves as input to the text encoder. Simultaneously, image data are processed through a pre-trained visual encoder and a Region Proposal Network (RPN). The RPN generates bounding box proposals, and the visual encoder aligns region features with corresponding textual features. Region of Interest (RoI) pooling is then applied to extract classification and localization information.

To further enhance the performance of the model, a Concept Enhancement Module (CEM) is introduced. The CEM employs cross-attention to weight text features based on their relevance to the image, effectively integrating visual and semantic information. Additionally, a temperature parameter is introduced to amplify the results of cosine similarity; the learnability of this parameter enables continuous weighting transformations of image features, further enhancing their representational power in the feature space. This strengthens the role of image features within the model. By combining the advantages of visual and textual data, our proposed framework provides a powerful and effective solution for wildlife conservation and monitoring, especially in challenging scenarios. This section describes the detailed structure of CECS-CLIP.

#### 3.2.1. Baseline Model

Our framework is implemented on the basis of the popular two-stage detector, faster R-CNN. The object detector first generates candidate regions using a Region Proposal Network (RPN), followed by further classification and regression of these candidate regions. Specifically, in the first stage, a series of candidate regions likely containing objects are extracted from the input image by the RPN. These candidate regions are then projected onto the feature map, and high-level features are extracted using a visual encoder. In the second stage, these features are fed into the ROI heads for target classification and bounding box regression, thereby precisely determining the target’s category and position. Through this two-stage process, more accurate detection and recognition of target objects in rare wildlife images are achieved.

#### 3.2.2. Visual Representation

**Figure 3:**
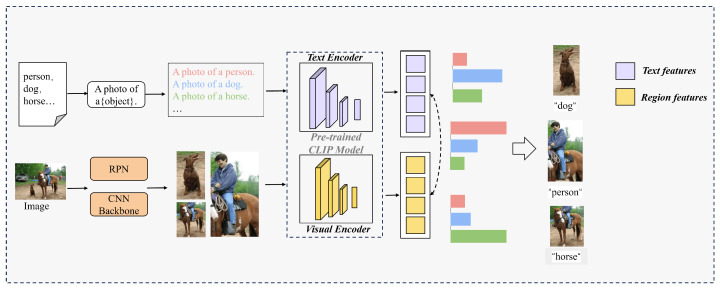
Visual–linguistic pretraining: Transforming image–text pairs into region–text pairs using the pretrained CLIP language encoder and Region Proposal Network (RPN) for image region alignment.

Pre-training of the visual encoder: Direct application of the CLIP model to object detection within image regions yields suboptimal results due to its inherent focus on image–text alignment at the holistic level rather than at a granular region-level. To address this limitation, we propose an enhanced region–text alignment approach, extending CLIP’s capabilities to learn region-level visual features and establish fine-grained correspondences between image regions and textual concepts.The pretraining of the visual encoder is shown in Figure 3.

Specifically, the pre-trained CLIP model was directly utilized as a teacher model, encompassing the teacher’s visual encoder $V_{t}$ and language encoder *L*. The visual encoder $V_{t}$ extracted the feature representation of the image *I* as $f_{I}=V_{t}\left(I\right)$, while the language encoder *L* extracted the feature representation of the text *j* as $g_{j}=L\left(j\right)$. The similarity $s_{ij}$ was computed as the dot product of the image and text features:

(1) $$s_{ij}=\frac{f_{I}\cdot g_{j}}{∥f_{I}∥∥g_{j}∥}$$

while minimizing the similarity between mismatched pairs. The softmax function was employed to transform the similarity into a probability, indicative of the match between the image region and text:

(2) $$P\left(f_{I},g_{j}\right)=\frac{\exp\left(\frac{s_{ij}}{\tau }\right)}{\sum_{k=1}^{N}\exp\left(\frac{s_{ik}}{\tau }\right)}$$

where τ is the temperature parameter, and *N* is the batch size. The contrastive learning loss function comprised two parts: one representing the probability of a match from image regions to text, and the other representing the probability of a match from text to image regions. Maximizing these probabilities facilitates improved alignment of image regions with text by the model. The loss function is expressed as follows:

(3) $$L_{\mathrm{contrastive}}=-\frac{1}{N}\sum_{i=1}^{N}\left(\log\frac{\exp\left(\frac{s_{ii}}{\tau }\right)}{\sum_{j=1}^{N}\exp\left(\frac{s_{ij}}{\tau }\right)}+\log\frac{\exp\left(\frac{s_{ii}}{\tau }\right)}{\sum_{j=1}^{N}\exp\left(\frac{s_{ji}}{\tau }\right)}\right)\;$$

The objective of the distillation loss is to harmonize the output of the student model with that of the teacher model, facilitating the transfer of knowledge throughout the pre-training phase. The pre-trained CLIP model, serving as the teacher model, computes the similarity $s_{i}^{\mathrm{teacher}}$ between image regions and text pairs, whereas the student model, represented by the image encoder in RegionCLIP, computes the similarity $s_{i}^{\mathrm{student}}$ for the same pairs. The distillation loss function is expressed as $L_{\mathrm{distillation}}=\sum_{i=1}^{N}{\left(s_{i}^{\mathrm{teacher}}-s_{i}^{\mathrm{student}}\right)}^{2}$. By minimizing the discrepancy between the teacher and student model outputs, the student model acquires the knowledge of the teacher model, enhancing its ability to achieve a region-level image encoder *V*.

Feature extraction from regions: The set of image regions $R=\{r_{1},r_{2},\ldots ,r_{n}\}$ was produced by the RPN, where each $r_{n}$ is a preprocessed image region. After feature extraction by the pre-trained image encoder *V*, the feature representations of all image regions were aggregated, forming the feature representation set, $V=\{v_{1},v_{2},\ldots ,v_{n}\}$.

#### 3.2.3. Textual Representation

Knowledge base construction for experts: To facilitate precise description and differentiation of object classes within photographs, the advanced language model GPT-3 is utilized to autonomously construct and refine the suite of visual feature descriptors for each category. These descriptors encapsulate a range of visual attributes pertinent to object classes, including color and shape, constituting critical components in the object detection process. Our method first utilized the generative capabilities of the large language model to produce a set of detailed visual feature descriptions for each class. These descriptions also extend to more subtle characteristics such as distinctive features of specific species, like the upright tail of the Tibetan macaque. By enriching and refining the descriptors for each class, the model is ensured to capture the subtle differences necessary to distinguish between different object classes. For example, in the case of the Tibetan macaque, not only are its fur color and body size described, but special emphasis is also placed on its upright tail: a unique biological trait that aids in improving detection accuracy and distinction in complex field environments. Additionally, our method includes an attribute association step that links the generated descriptors with the detected object instances in the images. This step further improves the accuracy of object detection by enhancing the model’s ability to recognize features of specific object classes.

Text encoding: To extract features from the target objects, the text encoder of the CLIP model was employed. The CLIP model, pre-trained on large-scale image–text pairs, was capable of capturing rich semantic information. In this study, a dataset containing *N* classes was defined, and using expert knowledge or species encyclopedias, the appearance descriptions for each species were extracted and encoded into description vectors $S_{N}=\left\{s_{1},s_{2},\ldots ,s_{N}\right\}$. Specifically, for the *n*th species, a description vector $s_{n}$ was constructed, which included descriptions of features such as fur color and body size. Simultaneously, species label vectors $C_{N}=\left\{c_{1},c_{2},\ldots ,c_{N}\right\}$ were defined, where each $c_{n}$ is a binary vector representing the class label of the *n*th species.

To integrate the description information and species labels, a knowledge vector $E_{K}$ was constructed by combining the description vectors and species label vectors. The knowledge vector was constructed using simple vector addition as follows: $E_{N}=S_{N}+C_{N}=\left\{e_{1},e_{2},\ldots ,e_{N}\right\}$. Here, each $e_{n}=s_{n}+c_{n}$ is the comprehensive feature representation for the *n*th species, combining appearance descriptions and class label information.

Next, the knowledge vectors $E_{N}$ were input into the CLIP text encoder *L* to obtain the high-level semantic feature representations for each species, $T=L(E_{N})$. Thus, a feature set *T* was obtained, encompassing the semantic features of all species in the dataset, which could then be utilized for the object detection task.

#### 3.2.4. Inter-Modal Interactions

**Figure 4:**
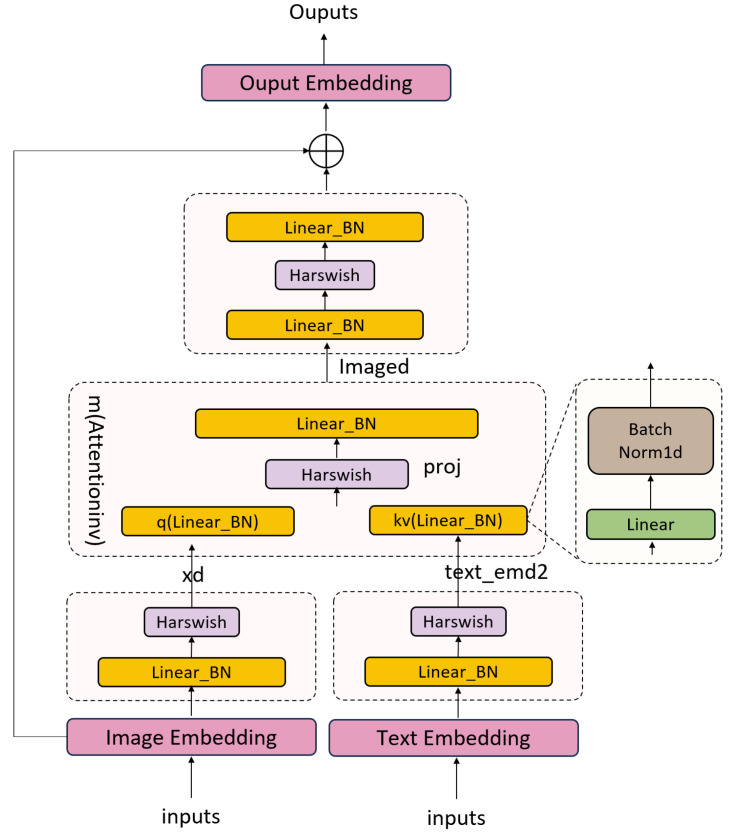
Network structure of concept enhancement module.

Concept enhancement: A cross-modal attention-based deformable block was incorporated into the backbone network, with its core function being to significantly enhance image feature recognition accuracy through the fusion of cross-modal information. By integrating textual information, this module not only augments the semantic expression of image embeddings but also effectively considers the semantic relationships between different concepts and their interactions with image embeddings. During the textual feature weighting process, texts more closely related to the image features are assigned higher weights, and the weighted textual features are then fused with the image features to obtain enhanced image features. Through the cross-attention mechanism, the deep integration of image and textual features is achieved, resulting in an enhanced image feature representation. This fusion ensures that the image features not only retain the original visual information but also incorporate the semantic information from the textual features.The specific network architecture is shown in Figure 4.

The textual feature *T* represents the original textual information acquired by the model, capturing the intrinsic semantics of the text data. The image feature *V* represents the features extracted from images, which encompass visual information that can complement textual information. The cross-attention mechanism CA serves as a pivotal component used to fuse features from disparate modalities. It dynamically modifies feature weights by computing the correlation between image features *V* and textual features *T*, thereby enhancing the text parts closely related to the image. The feedforward network FFN is a module composed of two linear layers, in accordance with the design principle of residual connections. It learns the residuals between input and output, aiding the network in better capturing complex patterns and reducing the risk of overfitting. In this way, the CEM not only enhances image features but also improves the model’s representational and generalization capabilities through the FFN.

(4) $$V_{\mathrm{aug},i}^{\prime }=V+\mathrm{CA}\left(W_{q}V,W_{k}T_{i},W_{v}T_{i}\right)$$

(5) $$\left[V_{\mathrm{aug},i}^{\mathrm{bas}},V_{\mathrm{aug},i}^{\mathrm{cap}}\right]=V_{\mathrm{aug},i}^{\prime }+\mathrm{FFN}\left(V_{\mathrm{aug},i}^{\prime }\right)$$

In our approach, the original image feature *V* is first enhanced through a cross-modal attention module. This module leverages the interaction between textual features $T_{i}$ and image features *V*, accomplished through the weighted sum of query $W_{q}V$, key $W_{k}T_{i}$, and value $W_{v}T_{i}$. The enhanced image feature $V_{\mathrm{aug},i}^{\prime }$ is composed of the sum of the original feature *V* and the cross-modal attention output. Subsequently, $V_{\mathrm{aug},i}^{\prime }$ is processed by a feedforward neural network (FFN) for further nonlinear transformation, resulting in a new set of features $V_{\mathrm{aug},i}^{\mathrm{cap}}$. These features are amalgamated with the original enhanced features $V_{\mathrm{aug},i}^{\mathrm{bas}}$ to form the final multimodal fused feature representation.

Continuous feature scaling: During the training phase, the visual encoder employs a contrastive learning strategy to align positive visual encodings with textual encodings within the same feature space without imposing any constraints on vector length. Therefore, to avoid errors in similarity computation due to varying feature vector lengths, it is necessary to perform scale normalization before calculating their similarity. In this case, the L2 norm is used for standardization, mapping the feature vectors $F_{I}$ and $F_{T}$ onto a unit hypersphere to standardize the scales of different feature vectors. The specific process is as follows:

(6) $$V=\{v_{1},v_{2},\ldots ,v_{n}\}$$

(7) $$V_{\mathrm{norm}}=\left\{\frac{v_{1}}{{∥V∥}_{2}},\frac{v_{2}}{{∥V∥}_{2}},\ldots ,\frac{v_{n}}{{∥V∥}_{2}}\right\}$$

where

(8) $${∥V∥}_{2}=\sqrt{\sum_{i=1}^{n}v_{i}^{2}}$$

The same normalization process is applied to the textual features. The normalized image and textual features are then used to compute cosine similarity, which can be simplified as an inner product similarity computation:

(9) $${\mathrm{sim}}_{c}os\left(V,I\right)=\frac{V\cdot I}{{∥V∥}_{2}{∥I∥}_{2}}={∥V∥}_{n}orm\cdot {∥I∥}_{n}orm$$

Typically, contrastive learning-based methods use the softmax function to convert image–text similarity predictions into class probabilities:

(10) $$p\left(y=i|x\right)=\frac{e^{\left(\frac{\cos(t_{i},f)}{\tau }\right)}}{\sum_{j=1}^{K}e^{\left(\frac{\cos(t_{j},f)}{\tau }\right)}}$$

(11) $$P_{i}=\left\{p\left(y=1|x\right),p\left(y=2|x\right),\ldots ,p\left(y=N|x\right)\right\}$$

where the hyperparameter is a temperature coefficient with a value range of (0,1), used to amplify the cosine similarity results and enhance the separation of prediction outcomes. The use of a distillation model increases the operational flexibility of this process. To further enhance the accuracy of the prediction results, a learnable parameter, Scale_factor (denoted as α), is introduced to replace it, allowing for continuous weighted transformation of image features to further strengthen their representation in the feature space:

(12) $$\alpha \leftarrow \alpha -\eta \frac{\partial L}{\partial \alpha }$$

(13) $$V_{\mathrm{scaled}}=\frac{1}{\alpha }\cdot V_{\mathrm{norm}}$$

#### 3.2.5. Object Detector

Object detection: The candidate regions $R=\{r_{1},r_{2},\ldots ,r_{n}\}$ generated by the RPN are projected onto the feature map, resulting in feature vectors $f_{i}$ for each region. These feature vectors are further processed by a pre-trained region-level visual encoder, generating higher-level feature representations $V_{\mathrm{scaled}}=\left\{v_{\mathrm{scaled}1},v_{\mathrm{scaled}2},\ldots ,v_{\mathrm{scaled}n}\right\}$. The features *V* are then fed into ROI heads, which consist of two subnetworks: a classification subnetwork and a regression subnetwork. The classification subnetwork outputs the class probability distribution $P=\{p_{1},p_{2},\ldots ,p_{n}\}$ for each region, while the regression subnetwork outputs the bounding box regression parameters $B=\{b_{1},b_{2},\ldots ,b_{n}\}$ for each region. This process extracts the target’s class and location from the input image, accomplishing the object detection task.

Loss function: In our approach, the overall loss function comprises three components, which can be expressed as follows:

(14) $$L_{\mathrm{loss}}=L_{\mathrm{contrastive}}+L_{\mathrm{cls}}+L_{\mathrm{reg}}.$$

The image encoder incorporates a contrastive learning loss, while the object detector includes a classification loss and a bounding box regression loss. To ensure accurate bounding box prediction, a bounding box regression loss is employed. This can be mathematically expressed as follows:

(15) $$L_{\mathrm{contrastive}}=-\sum_{i,j}\log\frac{e^{\left(\frac{\mathrm{sim}(f_{i},g_{j})}{\tau }\right)}}{\sum_{k}e^{\left(\frac{\mathrm{sim}(f_{i},g_{k})}{\tau }\right)}}$$

where $f_{i}$ denotes the feature representation of image region *i*, $g_{j}$ represents the feature representation of text description *j*, sim(·,·) denotes the similarity function between features, and τ is the temperature parameter controlling the smoothness of the softmax distribution.

The classification loss uses a cross-entropy loss function to compute the difference between each ROI’s class prediction and the ground-truth labels. The cross-entropy loss function is formulated as follows:

(16) $$L_{\mathrm{cls}}=-\sum_{c=1}^{C}y_{c}\log\left(p_{c}\right)$$

The classification loss, denoted as $L_{cls}$, is computed using the cross-entropy loss function. Here, *C* represents the total number of classes, and $y_{c}$ is the one-hot encoded true label. If a sample belongs to class *c*, then $y_{c}=1$; otherwise, $y_{c}=0$. The model’s predicted probability that the sample belongs to class *c* is denoted by $p_{c}$. If the predicted probability $p_{c}$ is close to the true label $y_{c}$, the loss will be small. Conversely, if the prediction probability deviates from the true label, the loss will be larger, incentivizing the model to adjust its parameters to reduce this error. The cross-entropy loss function is applied to each Region of Interest (ROI), and the total classification loss for the entire batch is obtained by summing the classification losses across all ROIs. For the regression loss, we employ the loss function, which combines the benefits of both L1 and L2 loss functions. It is formulated as follows:

(17) $$L_{\mathrm{reg}}=\sum_{i\in \{x,y,w,h\}}\left(\frac{|\Delta_{i}{|}^{2}}{2}\cdot ⊮(|\Delta_{i}\left|<1)+\right|\Delta_{i}\left|\cdot ⊮(\right|\Delta_{i}\left|\geq 1\right)\right)$$

where $\Delta_{i}$ represents the difference between the predicted and ground truth bounding boxes in terms of the center coordinates (x,y) and the width and height (w,h).

## 4. Experiments

### 4.1. Evaluation Metrics

We employed a robust set of evaluation metrics to assess the proposed model. The Average Precision (AP) is calculated at a confidence threshold of 0.5, which measures the model’s ability to identify objects at a moderate confidence level. Additionally, the mean Average Precision (mAP) is computed across an IoU threshold range from 0.5 to 0.95 with an increment of 0.05, allowing us to meticulously evaluate the model’s overall performance under varying strictness levels of the IoU criteria. To further refine our assessment, we defined “small”, “medium”, and “large” objects based on the area or pixel proportion they occupy in the image. Consequently, we introduce size-specific average precisions for small objects (APs), medium-sized objects (APm), and large objects (APl) to gain a more precise understanding of the model’s strengths and limitations when dealing with objects of different sizes.

In our evaluation, True Positives (TPs) represent the number of samples correctly predicted as the positive class, while False Positives (FPs) denote the number of samples incorrectly predicted as the positive class. False Negatives (FNs) are the samples that are actually positive but were incorrectly predicted as the negative class. Precision (P) is the ratio of TPs to the sum of TPs and FPs, and Average Precision (AP) is the mean of the precision values at different recall levels, which measures the area under the Precision–Recall (PR) curve. The AP is calculated using the following formula:

(18) $$P=\frac{TP}{TP+FP}$$

(19) $$R=\frac{TP}{TP+FN}$$

(20) $$AP=\sum_{k=1}^{n}\left(R_{k}-R_{k-1}\right)\cdot P_{k}$$

where $R_{k}$ is the recall at the k-th, threshold and $P_{k}$ is the precision at the k-th threshold. The mean Average Precision (mAP) at an IoU threshold of 0.5 for all categories is denoted as mAP, and it is calculated using the following formula:

(21) $$mAP=\frac{1}{N}\sum_{i=1}^{N}AP_{i}$$

where *N* is the number of categories. The mAP50:95 requires the model to maintain a high accuracy and reliability under stricter matching criteria, making it more challenging to achieve than mAP at a single IoU threshold.

For the YOLO series of single-stage object detection models, several metrics are included, such as precision (P), detection rate (DR), false positive rate (FPR), miss rate (MR), mAP50 (mean average precision at an IoU of 0.5), and mAP50:90 (mean precision at IoU thresholds ranging from 0.5 to 0.95), to more finely reflect the model’s performance at different IoU thresholds, particularly in terms of localization accuracy.

### 4.2. Model Performance Evaluation

The experimental framework extends beyond standalone testing of the proposed methodology to include a comparative analysis with state-of-the-art object detection algorithms. The comparative trials involved renowned single-stage object detection algorithms from the YOLO family and the esteemed two-stage fast R-CNN algorithm, both demonstrating exceptional performance across various public datasets. Additionally, experimentation with multimodal object detection models sharing the same backbone architecture—RegionCLIP, Detic, DetPro, and PromptDet—was conducted, with nearly identical parameter settings achieved in all cases.

The experimental results indicate that, on the dataset of rare and endangered wildlife, the multimodal object detection algorithm achieved a precision of 95.2%, representing an approximate improvement of 0.3% over the baseline models of the YOLO series. Compared to existing multimodal object detection algorithms, the model showed at least a 25% improvement in AP and a 15% improvement in AP50. On the more challenging web dataset, our method’s precision was comparable to the highest values of the YOLO series’ baseline models, achieving at least a 14% improvement in AP and 3.8% in AP50 over existing multimodal object detection algorithms.

To present these comparative results clearly, the AP and AP50 metrics of each model on both datasets are detailed in Table 1. Synthesizing these data, the multimodal object detection algorithm not only exhibits theoretical innovation but also demonstrates significant performance advantages in practical applications.

Based on the comparative analysis of Table 2 and Table 3, our method achieved a detection precision (P) of 95.2%, representing a 0.3% improvement over the YOLOv7s model, the most accurate in the YOLO series. This improvement signifies the superiority of our method in object detection tasks. In terms of the three key performance indicators—Detection Recall (DR), False Positive Rate (FPR), and Miss Rate (MR)—although there was a slight increase in our method’s Miss Rate (MR), it achieved the lowest False Positive Rate (FPR), indicating that our method is more effective in reducing false detections. This feature is crucial for object detection systems, especially in scenarios that demand high accuracy. Furthermore, on the more challenging web dataset, our method outperformed the others across all evaluation metrics. Considering all factors, our method demonstrates a clear advantage in improving detection accuracy and reducing false positives.

**Figure 5:**
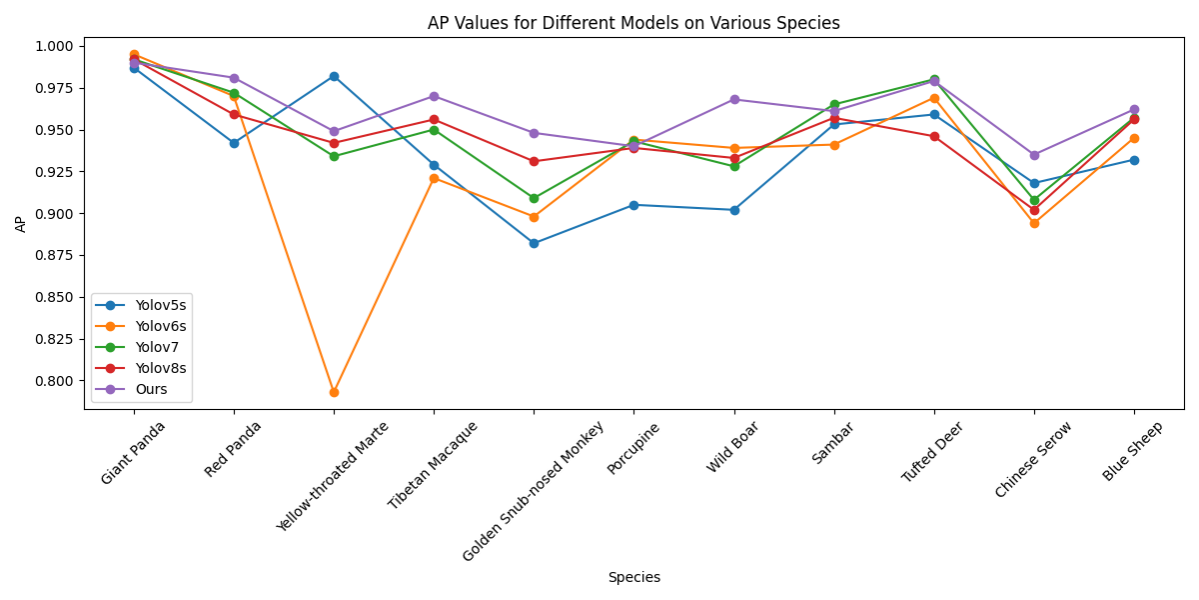
Average precision results of CECS-CLIP and other models on the wild dataset by species.

**Figure 6:**
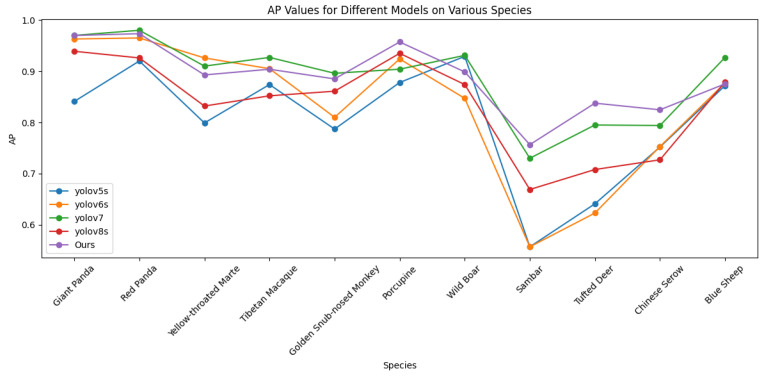
Average precision results of CECS-CLIP and other models on the web dataset by species.

We conducted a detailed performance evaluation of object detection for 11 species of wild animals, including the Giant Panda (Ailuropoda melanoleuca), Red Panda (Ailurus fulgens), and Yellow-throated Marten (Martes flavigula). To visually present the detection accuracy of these species, the corresponding Average Precision (AP) values are plotted in Figure 5 and Figure 6. The experimental results indicate that, on the wild dataset, the method achieved significantly high precision in detecting species such as the red panda, yellow-throated marten, Tibetan macaque (Macaca thibetana), and golden snub-nosed monkey (Rhinopithecus roxellana). Specifically, the precision for the yellow-throated marten reached 98.2%, showing an improvement of at least 3.3% over the YOLO series models. A comparative analysis revealed that the method significantly outperformed the YOLO series models in AP values for these species, demonstrating superior performance under complex backgrounds and low-light conditions.

On the more challenging web dataset, the method also showed high precision in detecting species such as the Tibetan macaque, golden snub-nosed monkey, and porcupine (Erethizon dorsatum). For instance, the method achieved an AP of 95.7% for porcupine detection, representing a 2.2% improvement over the YOLO series models. These results further validate the accuracy of the algorithm across different datasets and environmental conditions. Although the detection accuracy for some species in the multimodal object detection model did not reach the highest value, overall, the method made significant progress in improving the detection accuracy of species that were poorly detected by the baseline models.

The detailed experimental results and comparative analysis are presented in Figure 6, which displays the AP values of different species in the two datasets and compares them with the YOLO series models. These results not only demonstrate the effectiveness of the multimodal object detection algorithm but also provide important references for future applications in wildlife monitoring.

**Figure 7:**
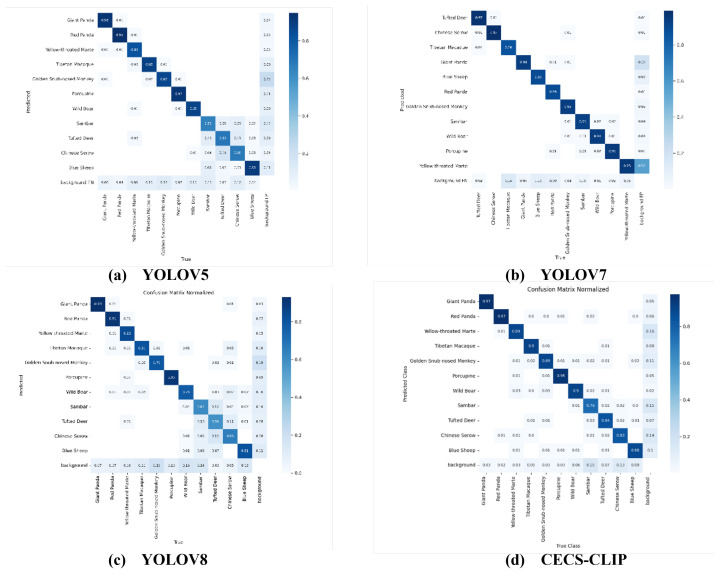
Confusion matrix of CECS-CLIP and the other models on the wild dataset.

In this study, CECS-CLIP was employed to detect multiple object categories in images. As shown in Figure 7, the confusion matrix provides a comprehensive understanding of the model’s performance across different categories.

From the matrix, it can be observed that the model performed excellently in detecting categories such as giant panda, red panda, and porcupine, achieving accuracies of 97%, 97%, and 95%, respectively. The detection accuracy for Sambar was slightly lower, at only 76%. This difference may be attributed to the lower frequency of Sambar appearances in the images, resulting in insufficient samples during model training. Additionally, the recall rate for Chinese serow was relatively low, indicating that the model missed a significant number of true samples in this category. This could be due to the similarity of Chinese serow features to those of other categories, making it difficult for the model to distinguish between them. Despite a few misclassifications in minority categories, the overall performance of the model in classifying the primary categories remained commendable, demonstrating its exceptional capability in recognizing and distinguishing between key species. This superior performance is not only reflected in the high accuracy but also in the model’s ability to effectively handle background noise, enhancing its reliability and effectiveness in practical applications.

**Figure 8:**
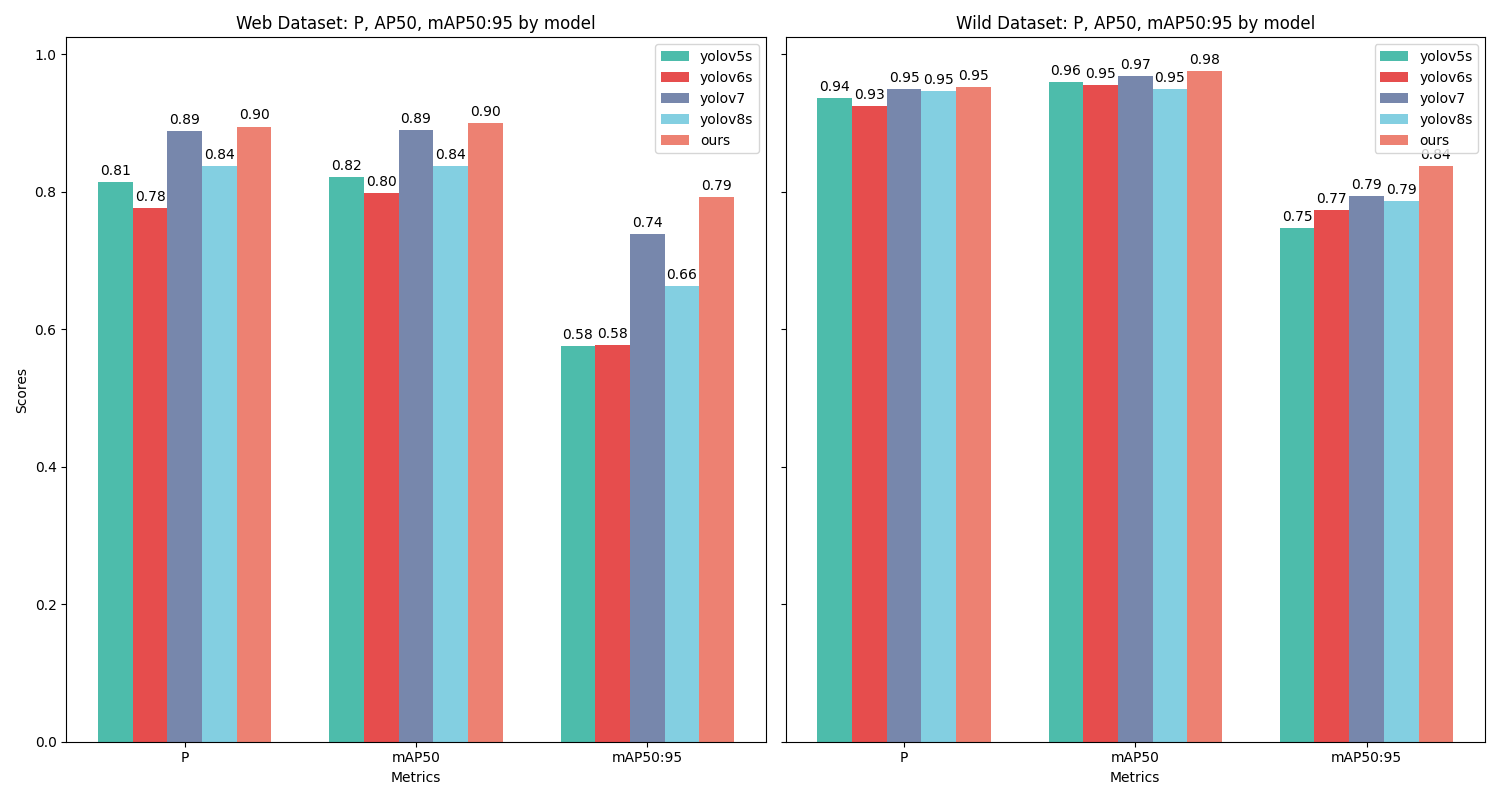
Experimental results of CECS-CLIP and other models based on P, mAP50, and mAP50:90 metrics across the web and wild datasets.

Comparative experiments against classic models such as the YOLO series demonstrate a significant advantage of the method in detecting small targets. On the wild dataset, the approach achieved an accuracy of 84% in the mAP50:90 metric, surpassing the YOLO series models and highlighting the algorithm’s efficiency in handling small-sized targets. On the web dataset, despite facing more complex backgrounds and target sizes, the method achieved a mAP50:90 accuracy of 79%, demonstrating superior performance compared to the comparative models. These results confirm the effectiveness of the method in small target detection and indicate its potential for practical applications. Particularly in rare wildlife monitoring, the method significantly enhances the detection accuracy of small-sized targets, providing more reliable technical support for wildlife conservation and research. Figure 8 presents a detailed comparison of the mAP50:90 metric between the method and the YOLO series models on the two datasets, further confirming the superiority of the method in small target detection.

Further in-depth analysis of the performance in small target detection focuses on the following key metrics: average precision on small targets (APs) and average precision on medium targets (APm). These metrics provide a nuanced reflection of the model’s detection capabilities across different target sizes, which is crucial for monitoring rare wildlife where accurate identification of small targets is essential.

The experimental results indicate that, in terms of the APs metric, the method achieved a precision of 28.7%, and for the APm metric, it achieved a precision of 52.7%. These figures highlight the superiority of the method in small target detection and demonstrate its high accuracy in detecting medium-sized targets. Compared to existing models, the method shows significant performance improvements in these metrics, further proving the effectiveness and robustness of the algorithm in handling small-sized targets. Detailed experimental results are presented in Table 4.

This study aimed to perform an in-depth analysis of the contributions of each key component in the proposed multimodal object detection algorithm. To achieve this, a series of ablation experiments were designed and implemented. Using the baseline model (RegionCLIP) as a control, models were constructed to include only the Concept Enhancement (CE) module, only the Continuous Feature Scaling (CS) module, and the CECS-CLIP model proposed in this paper for comparative analysis. The core purpose of these experiments was to systematically evaluate the specific impact of key technologies, such as the concept enhancement module and the continuous weighted feature smoothing module, on the overall performance of the model. The improved models were compared with the baseline model on the wild dataset. The evaluation metrics included Average Precision(AP), Average Precision on small targets (APs), Average Precision on medium targets (APm), and Average Precision on large targets (Apl).

The experimental results indicate that the introduction of both the concept enhancement module and the continuous weighted feature smoothing module significantly enhanced the model’s detection performance. The model with only the CE module demonstrated significant improvements in metrics such as AP, AP50, and APm. This indicates that the CE module plays a key role in enhancing the model’s detection capabilities for small targets. The model with only the CS module also demonstrated enhancements in metrics such as AP, AP50, and APm. The CS module, by normalizing features, enhanced recognition capabilities for medium-sized targets. The CECS-CLIP model, which combines the CE and CS modules, achieved optimal performance across all the evaluation metrics. Notably, its AP, AP50, APm, and Apl metrics were significantly higher than those of the variants with a single module, indicating that the synergistic effect of the two modules greatly enhances overall detection performance. To more intuitively demonstrate these improvements, each improvement method and its corresponding experimental data are listed in Table 5.

Furthermore, experimental explorations were conducted to determine the impact of different scaling factors on the model’s Average Precision (AP) and AP50 precision within the feature smoothing (CS) module. The study of the generalization capability of multimodal object detection models revealed that RegionCLIP could be effectively applied to new domains with only minor adjustments. Fine-tuning experiments on the RegionCLIP model revealed the significant influence of the number of iterations on model performance. Specifically, within the range of 10,000 to 30,000 iterations, the model’s accuracy continued to improve. After 30,000 iterations of training, the model’s average precision (AP) reached 55.86%, and the AP50 metric, i.e., under the condition of a recall rate exceeding 50%, increased significantly to 85.73%.

Further research revealed that adjusting the model’s scaling factor significantly impacts performance. The specific results are displayed in Table 6. The experiments showed that when the scaling factor was set to 0.9, compared to 0.5, the model’s AP value increased by approximately 28%, and the AP50 increased by about 35%. Based on this finding, an improved method was proposed that, by optimizing the scaling factor, achieved a model performance comparable to that of the RegionCLIP model after 30,000 iterations, even with only 10,000 iterations in some cases. Based on the experimental results, the scaling factor was set to 0.9 and adopted as the standard setting for subsequent experiments.

To thoroughly analyze the impact of training iteration counts on model performance and duration, a series of controlled experiments were designed and executed, as detailed in Table 7. The experimental results are listed in the table. The table reports the time required for model training under different iteration settings, as well as the values for average precision (AP) and AP50. Detailed analysis of the tabular data reveals that within 30,000 iterations, the average precision of the model showed a steady upward trend with increasing iteration counts. However, for single-modal and multimodal object detection tasks, achieving optimal performance requires careful selection of the number of training iterations based on task characteristics and requirements. This suggests that in practical applications, balancing model performance with training costs is necessary to achieve an optimal combination of efficiency and effectiveness.

In the practice of wildlife monitoring, the use of trap cameras often results in empty shots, where the captured images do not contain any animals. To assess the model’s performance in handling such images, we specifically selected a batch of empty images for testing. These images were submitted to the model for analysis, and subsequently, we employed manual review to verify the model’s recognition results. The experimental results are presented in Table 8.

Following a meticulous manual assessment of our collected image dataset, comprising 249 targets across 10 distinct categories, our model successfully identified 214 targets, yielding an impressive accuracy rate of 85.9%. Notably, the recognition accuracy for the sambar was relatively low, at 53.3%, whereas it achieved a remarkable 100% accuracy for the giant panda. Encouragingly, all other species exhibited accuracy rates surpassing the 70% threshold. Moreover, our model demonstrated an exceptional capability in filtering out blank images, with a success rate of 92% out of the 50 blank photos inspected. The experimental results show that the model can effectively identify empty images, with an `object absence detection’ accuracy rate of 92%. This result highlights the model’s efficient filtering capability when facing scenes without targets. The model did not mistakenly identify empty images as containing objects, which not only demonstrates its excellent robustness but also proves its reliability in practical applications. This capability is crucial for reducing false positives and improving monitoring efficiency, especially in wildlife reserves where it is necessary to distinguish between real animal activity and empty shots.

## 5. Discussion

In this study, we focus on leveraging multimodal object detection models to enhance the neural network’s recognition capabilities on the GPNP image dataset LoTE-Animal. This model integrates image data with textual information to supplement the context of the target objects, highlighting important areas by applying continuous weighted feature smoothing to feature maps, thereby enhancing the expressiveness of the feature maps and improving detection accuracy. To comprehensively evaluate the model’s performance, we conducted extensive experiments and compared them with state-of-the-art technologies in the current field. In this section, we will discuss in detail the experimental results, including the model’s performance on the LoTE-Animal dataset and its comparison with existing technologies. Additionally, we will explore the challenges the model may face in practical application scenarios, such as handling target objects under different environmental conditions and the robustness of detection when targets are small in size or partially occluded.

**Figure 9:**
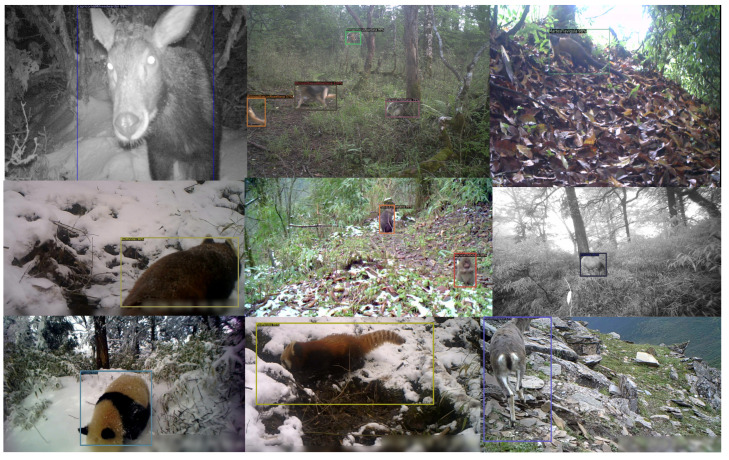
Partial results of our method generalization test.

The recognition results displayed in Figure 9 highlight the performance of the CECS-CLIP model under various lighting conditions, background complexities, and target sizes. These image examples include a variety of species of different sizes from the dataset, showing the model’s generalization capability in diverse scenarios. Based on these visual results, we can see the recognition accuracy of the CECS-CLIP model in actual national park environments, which is of significant practical importance for wildlife conservation and monitoring efforts.

**Figure 10:**
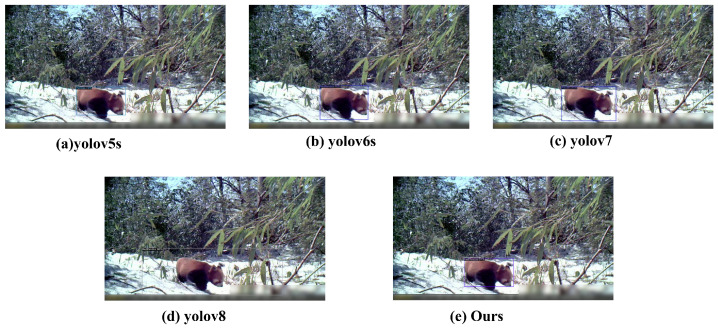
Experimental results of our method compared with other models.

**Figure 11:**
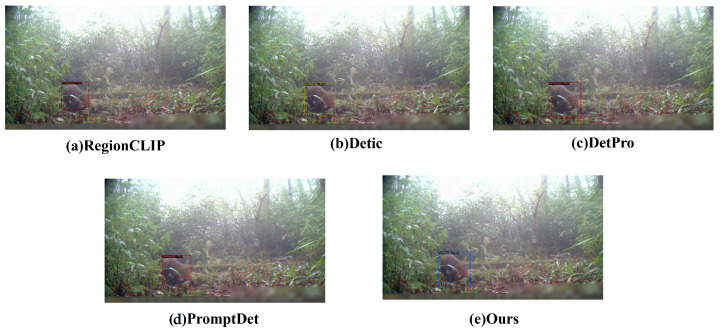
Experimental results of our method compared with other multimodal models.

To comprehensively evaluate the performance of our proposed multimodal object detection method (CECS-CLIP), we selected advanced multimodal object detection models that also utilize textual information, such as RegionCLIP, Detic, DetPro, and PromptDet, as baselines. These models have been proven to have high accuracy and robustness in the field of object detection. In the experiments, all models used nearly identical parameter configurations to ensure the fairness and accuracy of the comparison. Based on a careful analysis of the data presented in Figure 10, our method demonstrates significant superiority in the key evaluation metrics of Average Precision (AP) and AP50. The results of the comparative experiments are summarized in Figure 11. Notably, on the rare and endangered wildlife dataset, our method achieved an average accuracy of 95.8%, which an approximately 24% improvement over existing multimodal object detection techniques. This significant performance enhancement not only confirms the potential application of our model in the wild environment but also highlights its efficiency in detecting small targets in complex scenarios. We believe that this significant performance improvement is mainly attributed to the innovative Concept Enhancement Module (CEM) in our model and the Continuous Feature Scaling (CS) with an optimized scaling factor configuration. Through these technical improvements, our model can more effectively capture the visual content of images and achieve more accurate target localization in the feature space. The CEM enhances the model’s ability to recognize target categories by introducing textual information, while CS improves the model’s sensitivity to target shape and size by optimizing feature representation.

**Figure 12:**
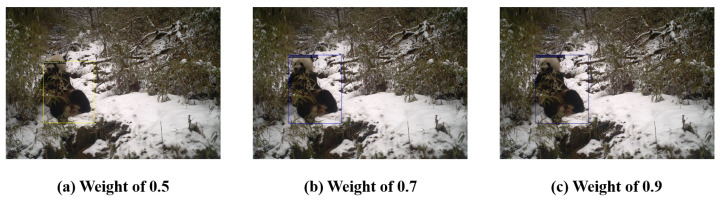
Comparative experimental results of different weights.

Furthermore, after incorporating the Continuous Feature Scaling (CS) algorithm into our experimental model, we observed improvements in both Average Precision (AP) and AP50. This study thoroughly explored the impact of different scaling factors on object detection performance and visually demonstrated the specific effects of various weight adjustments on detection accuracy and recall rates, as shown in Figure 12. This enhancement not only increased the training efficiency but also reduced the consumption of computational resources. The augmentation of cosine similarity outcomes significantly bolstered the model’s representational efficacy and discriminative capacity within the feature space. An analysis of the experimental data indicates that there are significant differences in the impacts of different parameters on model performance.

In this study, to gain an in-depth understanding of the contributions of various key components in our proposed multimodal object detection algorithm, we designed and conducted a series of ablation experiments. These experiments specifically focused on the impact of the Concept Enhancement Module (CEM) and the Continuous Feature Scaling (CS) module on model performance. The results are summarized in Figure 12, illustrating the precision performance of the different variants.

Through meticulous data analysis, we found that by simply introducing animal morphological features and environmental information, the addition of the Concept Enhancement Module (CEM) increased the model’s precision by 11%. This significant improvement indicates that integrating textual information greatly enhances the model’s ability to recognize targets. Furthermore, by incorporating the Continuous Feature Scaling (CS) module, we increased the model’s precision by an additional 7%. This result confirms the importance of introducing learnable parameters for the continuous weighted transformation of image features; a transformation that enhances the model’s representational power in the feature space and significantly improves its sensitivity to the morphological attributes and sizes of target entities.

**Figure 13:**
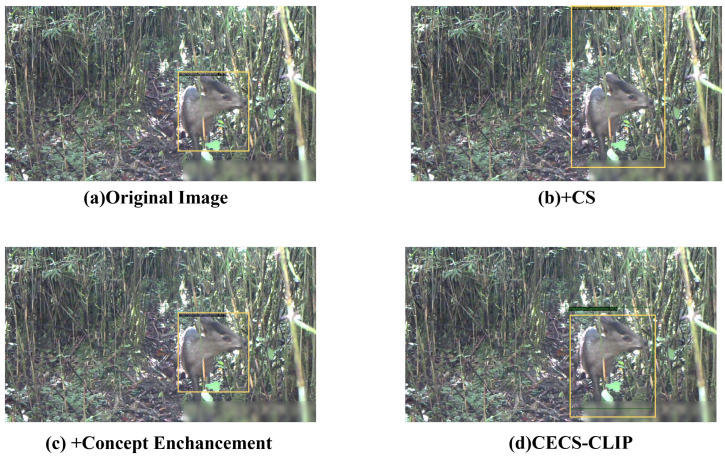
Visualization results of the improved model.

However, based on observations from Figure 13, we also noticed that in some cases, adding only the CS module may lead to inaccurate localization of the target detection box. This may be due to the process of amplifying cosine similarity results during feature normalization, where excessive scaling can lead to feature distortion or overemphasis on certain features, thereby interfering with the model’s correct understanding of the target’s shape and size, resulting in inaccurate bounding box localization and deviation from the true position of the target.

Our CECS-CLIP model, which combines the CE and CS modules, achieved the best performance across all the evaluation metrics. These results not only confirm the effectiveness of each component in our model but also reveal their synergistic effects. This synergy not only improves the model’s overall detection accuracy but also specifically enhances its ability to detect small targets. In complex scenes, the concept enhancement module assists the CS module in capturing target features more accurately by providing rich semantic information, thereby optimizing the localization of the detection box.

## 6. Conclusions

In this study, the primary challenge we faced was enhancing the recognition accuracy of small targets in images of rare and endangered wildlife, especially under adverse conditions such as environmental noise, complex backgrounds, and occlusions. To address these issues, we proposed a multimodal object detection algorithm that integrates textual information. This algorithm not only utilizes image data but also incorporates rich textual information, including category names, feature descriptions, and the living environments of the targets, thereby enhancing the model’s ability to recognize the objects. Through a series of experiments, we validated the effectiveness of the proposed algorithm. The experimental results indicate that, compared to existing single-modal object detection methods, our multimodal algorithm achieved significant performance improvements in evaluation metrics such as Average Precision (AP) and AP50. These improvements are primarily attributed to the introduction of textual information, which provides additional semantic guidance for the model, and the incorporation of the Continuous Feature Scaling (CS) method, which aids in locating and identifying targets that are difficult to confirm solely based on visual information due to their small size or partial occlusion.

Furthermore, we introduced the Continuous Feature Scaling (CS) algorithm, which includes trainable parameters to enhance the discriminative power of our predictive results. This algorithm applies a series of adaptive weighted transformations to image features, significantly enhancing their representational efficacy within the feature space. This contributes to the stability and robustness of the feature maps. We integrated the Concept Enhancement Module (CEM), which utilizes cross-attention to augment the original image features, further boosting the model’s capability for object detection. Our model achieved an AP of 95.8% and an AP50 of 97.6% on the LoTE-Animal dataset. Comparative experiments with classic YOLO models and multimodal object detection models showed that our model improved the precision value by 0.3% over the most accurate YOLOv7 and increased the mAP50:90 metric by 4.3%, indicating a significant enhancement in recognition accuracy. Compared with other multimodal object detection models, the APm and Apl metrics were improved by 25% and 0.8%, respectively.

Although our method performed well in multimodal object detection tasks, certain challenges and limitations were noted during the experiments. For example, under extreme conditions such as low lighting or high background noise, the model’s detection accuracy may be impacted. As some species’ detection accuracy is influenced by their occurrence frequency in images, future work will focus on increasing data sample diversity and optimizing the model structure. By collecting and integrating a more diverse set of data samples, the model’s performance across all categories will be enhanced. Future work will also focus on further optimizing the model to improve its performance under such complex conditions. In addition, further research will be conducted to improve the model’s generalization capabilities and explore its application to broader scenarios, such as drone surveillance and real-time video analysis.

## Figures and Tables

**Table 1:** Comparison of CECS-CLIP model with other models, including single-stage object detection models (FCOS), two-stage object detection models (Fast RCNN), novel object detection models (DETR, Sparse R-CNN, TOOD, DiffusionDet), and multimodal object detection models (RegionCLIP, DeTIC, DetPro, and PromptDet)—experimental results.

Model	Backbone	AP (web)	AP (wild)	AP50 (web)	AP50 (wild)
Faster R-CNN [51]	r50	0.554	0.698	0.851	0.931
	r101	0.588	0.710	0.863	0.933
FCOS [52]	r50	0.406	0.701	0.609	0.919
	r101	0.422	0.723	0.623	0.933
DETR [53]	r50	0.561	0.397	0.716	0.61
Sparse R-CNN [54]	r50	0.650	0.734	0.833	0.945
	r101	0.683	0.744	0.839	0.945
TOOD [55]	r50	0.685	0.751	0.851	0.938
	r101	0.696	0.758	0.856	0.941
DiffusionDet [56]	r50	0.707	0.761	0.865	0.941
	r101	0.698	0.762	0.85	0.954
RegionCLIP [45]	r50	0.696	0.559	0.825	0.776
Detic [57]	r50	0.721	0.544	0.846	0.743
DetPro [58]	r50	0.749	0.713	0.867	0.824
PromptDet [59]	r50	0.7	0.621	0.828	0.802
Ours	r50	0.889	0.958	0.905	0.976

**Table 2:** Performance metrics of YOLO and our method on the wild dataset.

Model	P	DR	FPR	MR
YOLOv5s [24]	0.936	0.922	0.068	0.078
YOLOv6 [60]	0.925	0.910	0.081	0.090
YOLOv7s [61]	0.949	0.925	0.054	0.075
YOLOv8s [62]	0.947	0.897	0.056	0.103
Ours	0.952	0.918	0.050	0.082

**Table 3:** Performance metrics of YOLO and our method on the web dataset.

Model	P	DR	FPR	MR
YOLOv5s [24]	0.814	0.763	0.229	0.237
YOLOv6 [60]	0.776	0.720	0.289	0.280
YOLOv7s [61]	0.888	0.820	0.126	0.180
YOLOv8s [62]	0.837	0.768	0.195	0.232
Ours	0.895	0.850	0.117	0.150

**Table 4:** Fine-grained metric experimental results of CECS-CLIP compared with other models: APs, APm, and APl.

Model	APs	APm	APl
YOLOv6	0.04	0.274	0.605
RegionCLIP	0.282	0.06	0.582
Ours	0.287	0.527	0.613

**Table 5:** Comparison of ablation experiments.

Model	AP	AP50	APs	APm	APl
RegionCLIP	0.559	0.776	0.282	0.06	0.582
RegionCLIP+CE	0.796	0.854	0.285	0.534	0.60
RegionCLIP+CS	0.856	0.891	0.281	0.462	0.59
RegionCLIP+CE+CS	0.958	0.976	0.287	0.527	0.613

**Table 6:** Comparative analysis of experimental results under different weight settings and comparative study with RegionCLIP fine-tuning method.

Weight Parameter	Iter	AP	AP50
0.5	10,000	0.214	0.5
0.7	10,000	0.382	0.694
0.9	10,000	0.574	0.859
RegionCLIP fine-tuning	10,000	0.296	0.596
RegionCLIP fine-tuning	20,000	0.422	0.728
RegionCLIP fine-tuning	30,000	0.559	0.776

**Table 7:** Comparative study of accuracy results and training time with different numbers of nodes.

Iter	AP	AP50
5000	0.462	0.79
10,000	0.573	0.859
15,000	0.694	0.892
20,000	0.737	0.929
30,000	0.958	0.976

**Table 8:** Statistics on the number of correct identifications of rare wildlife species and blank identifications in robustness testing.

Species	Actual	Correct Estimate	Correct Rate
Giant panda	35	35	1.000
Red panda	25	23	0.920
Tibetan macaque	28	24	0.857
Golden snub-nosed monkey	19	17	0.895
Porcupine	27	25	0.926
Tibetan macaque	20	18	0.900
Sambar	30	16	0.533
Tufted deer	18	16	0.888
Chinese serow	24	22	0.917
Blue sheep	23	18	0.783
All	249	214	0.859
Empty shots	50	46	0.92

## Data Availability

The authors do not have permission to share the data.
